# Association between fatty liver index and cardiovascular-renal-metabolic syndrome risk: Nonlinear relationship based on NHANES 2011–2020 and mediating effect analysis of TyG, SII, and SIRI

**DOI:** 10.1097/MD.0000000000046342

**Published:** 2026-05-12

**Authors:** Qinke Lv, Chun Yao, Jian Zhong

**Affiliations:** a The First Clinical Medical College, Graduate School of Guangxi University of Traditional Chinese Medicine, Nanning, China; b Guangxi University of Traditional Chinese Medicine, Nanning, China; c Department of Nephrology, The First Affiliated Hospital of Guangxi University of Traditional Chinese Medicine, Nanning, China.

**Keywords:** cardiovascular-kidney-metabolic syndrome, chronic kidney disease, fatty liver index, metabolic diseases, NHANES

## Abstract

The Fatty Liver Index (FLI) has been linked to several metabolic diseases, yet its relationship with cardiovascular-kidney-metabolic (CKM) syndrome remains uncertain. This cross-sectional study analyzed data obtained from the 2011–2020 National Health and Nutrition Examination Survey cycles. We employed multivariable-adjusted logistic regression models to examine the relationship between FLI and cardiovascular-kidney-metabolic (CKM) syndrome risk, complemented by comprehensive subgroup analyses. Restricted cubic spline (RCS) models with 3 knots were implemented to explore potential nonlinear associations between FLI and CKM syndrome. Furthermore, causal mediation analysis was conducted to assess the potential mediating roles of systemic immune-inflammation index (SII), systemic inflammation response index (SIRI), and triglyceride-glucose (TyG) index in the FLI-CKM syndrome association. In the fully adjusted model, a 1-unit increase in FLI was associated with an approximate 3% rise in CKM syndrome risk (adjusted odds ratio [OR] = 1.03, 95% confidence interval [CI]: 1.02–1.03, *P* < .001). Participants in the highest FLI quartile had an 18.1-fold higher risk of CKM syndrome compared to those in the lowest quartile (adjusted OR = 18.10, 95% CI: 11.70–27.90, *P* < .001). The RCS analysis confirmed a nonlinear relationship between FLI and CKM syndrome risk (*P* for non-linearity < .001, *P* for overall < .001). A threshold effect was identified, with 25.01 as the inflection point, after which the relationship between FLI and CKM syndrome risk leveled off. In addition, TyG, SII, and SIRI were identified as mediating factors between FLI and CKM prevalence, accounting for 18.82%, 0.77%, and 0.56% of the mediating proportions, respectively. The research findings emphasize the significant association between FLI and CKM prevalence, with TyG-related indicators playing a major mediating role.

## 1. Introduction

Cardiovascular-kidney-metabolic (CKM) syndrome is a complex disorder marked by the interplay of cardiovascular, renal, and metabolic dysfunctions. Given its association with elevated morbidity and mortality, CKM syndrome has become a significant public health concern. This syndrome encompasses chronic kidney disease (CKD), heart disease, diabetes, and metabolic disorders like obesity.^[1,2]^ The global prevalence of these conditions is on the rise, creating substantial burdens on healthcare systems and economies worldwide.^[3]^ In the United States, CKM syndrome contributes significantly to deaths related to cardiovascular and renal diseases, underscoring the urgent need for early detection and timely intervention.^[4]^ Understanding the risk factors associated with CKM syndrome is vital for devising preventive strategies and enhancing clinical outcomes.

The Fatty Liver Index (FLI) is a noninvasive scoring system designed to estimate the probability of nonalcoholic fatty liver disease (NAFLD) based on factors such as body mass index (BMI), waist circumference (WC), triglyceride levels, and gamma-glutamyl transferase (GGT).^[5]^ NAFLD is highly prevalent among individuals with metabolic syndrome and is associated with an increased risk of cardiovascular and renal diseases, recently, NAFLD has been redefined as metabolic dysfunction-associated steatotic liver disease, which emphasizes the central role of metabolic dysregulation.^[6,7]^ FLI serves as a useful tool for clinicians in identifying individuals at risk of developing fatty liver, a condition that often precedes various metabolic complications. Despite its widespread use in predicting liver disease, FLI’s role in assessing CKM syndrome risk remains largely unexplored.

Previous studies have demonstrated a strong relationship between FLI and several metabolic conditions, including type 2 diabetes, hypertension, and cardiovascular disease.^[8–12]^ However, the precise link between FLI and CKM syndrome remains uncertain. Further research is required to clarify whether FLI can reliably predict CKM syndrome across different populations and risk profiles.

This study aims to bridge this knowledge gap by analyzing data from the National Health and Nutrition Examination Survey (NHANES) to examine the relationship between FLI and CKM syndrome prevalence among U.S. adults. Specifically, the objectives of this study are to assess CKM syndrome prevalence across varying FLI levels, evaluate the consistency of this association across FLI quartiles and cardiovascular disease risk, and investigate potential moderating effects of subgroups such as age, sex, and comorbidities on the FLI-CKM syndrome risk relationship. Addressing these questions will provide a deeper understanding of FLI as a potential predictor of CKM syndrome risk and its relevance in clinical practice.

## 2. Materials and methods

### 2.1. Study population

**Figure 1.**
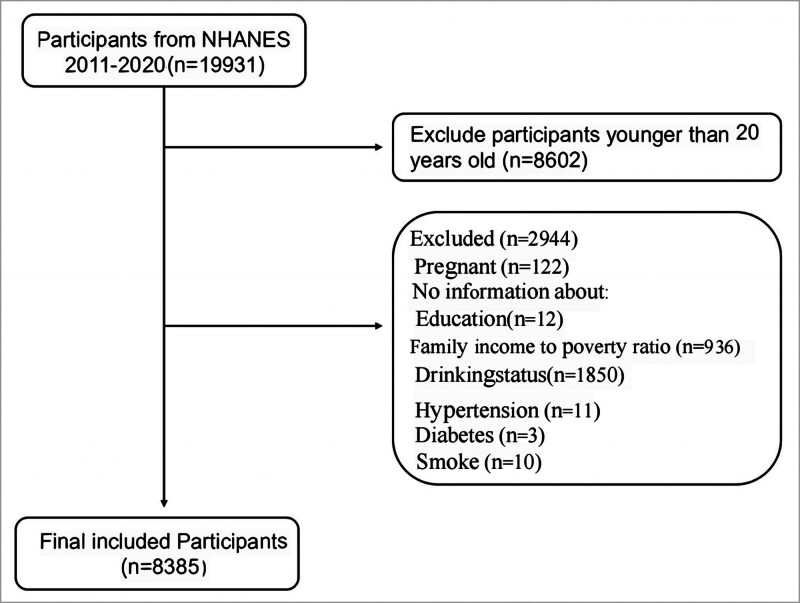
Flowchart of study participant inclusion and exclusion criteria.

The NHANES, conducted by the National Center for Health Statistics, aims to evaluate the health and nutritional status of the non-institutionalized civilian population in the United States. This study used data from NHANES participants between 2011 and 2020, initially including 19,931 individuals, of which 11,546 participants were excluded due to missing data. Therefore, 8385 participants were ultimately included (Fig. 1).

### 2.2. Outcome measurement

Currently, there is no universally accepted definition for CKM syndrome. Based on a review of existing literature, we defined CKM syndrome as cardiometabolic syndrome (CMS) occurring alongside CKD.^[13,14]^ The definition of CMS was derived from the National Cholesterol Education Program Adult Treatment Panel III (NCEP-ATP III) report, diagnosing CMS in individuals who met at least 3 of the following 5 criteria: central obesity (WC ≥ 102 cm for men or ≥ 88 cm for women); hypertriglyceridemia (serum triglycerides ≥ 150 mg/dL); low HDL cholesterol (serum HDL-c < 40 mg/dL for men or < 50 mg/dL for women); hypertension (systolic blood pressure ≥ 130 mm Hg, diastolic blood pressure ≥ 85 mm Hg, or receiving antihypertensive treatment); and hyperglycemia (fasting glucose ≥ 100 mg/dL or receiving antidiabetic treatment).^[15]^ Waist circumference, weight, and height were measured by trained NHANES personnel at the Mobile Examination Center, while systolic and diastolic blood pressure readings were recorded as the average of up to 4 measurements taken after a 5-minute rest.

CKD was defined as an estimated glomerular filtration rate of < 60 mL/min/1.73 m² or a urine albumin-to-creatinine ratio of > 30 mg/g.^[16]^ The estimated glomerular filtration rate was calculated using the CKD-EPI equation, developed by the Chronic Kidney Disease Epidemiology Collaboration in 2009.^[17]^

### 2.3. Exposure measurement

The study employed the FLI to evaluate participants’ obesity status. FLI was calculated using BMI, WC, TG, and GGT through the following formula^[18]^:


$$\begin{array}{l} FLI=\frac{e^{0.953\times ln\left(TG\right)+0.139\times BMI+0.718\times ln\left(GGT\right)+0.053\times WC-15.745}}{1+e^{0.953\times ln\left(TG\right)+0.139\times BMI+0.718\times ln\left(GGT\right)+0.053\times WC-15.745}}\times 100 \end{array}$$

BMI and WC were measured by trained professionals at the Mobile Examination Center. Triglyceride levels were analyzed at the University of Minnesota using a Roche Modular P biochemical analyzer, while GGT was measured on-site at the NHANES Mobile Examination Center using a Beckman Coulter DxH 800 instrument.

### 2.4. Mediator measurement

The indexes were calculated by the following formula.^[19,20]^


$$\begin{array}{l} TyG=ln\left(\frac{TG\times plasma\;glucose}{2}\right) \end{array}$$

$$\begin{array}{l} SIRI=\frac{\left(Neutrophilcount\times monocytecount\right)}{lymphocyte\;count} \end{array}$$

$$\begin{array}{l} SII=\frac{\left(\;plateletcount\times neutrophilcount\right)}{lymphocyte\;count} \end{array}$$

### 2.5. Covariates

Sociodemographic and lifestyle characteristics were collected as covariates, including age, sex, race/ethnicity (Mexican American, Other Hispanic, Non-Hispanic White, Non-Hispanic Black, Other Race), education level (<9th grade, 9–11th grade, high school diploma/GED, some college/AA degree, ≥college graduate), family income-to-poverty ratio, drinking status, smoking status, hypertension, and diabetes. Due to the high collinearity between BMI and FLI, they were not included as covariates. Hypertension was defined as a diagnosis of high blood pressure or current use of prescribed antihypertensive medication.^[21]^ Diabetes was defined as physician-diagnosed diabetes, a two-hour glucose (OGTT) level of ≥ 11.1 mmol/L, or a Fasting Glucose level of ≥ 7.0 mmol/L.^[21]^ Prediabetes was defined as a diagnosis of prediabetes, an OGTT level between 7.8 and 11.1 mmol/L, or a Fasting Glucose level between 6.1 and 6.9 mmol/L.^[21]^ Smoking status was categorized as nonsmokers (never smoked or quit smoking for over 1 year) and current smokers (currently smoking, smoked at least one day in the past 30 days, or smoked more than 2 cigarettes per day after quitting).^[21]^ Drinking status was classified as nondrinkers (fewer than 12 lifetime drinks) and current drinkers (at least 12 drinks per year or having consumed alcohol more than 6 times in the past 12 months).^[21]^

### 2.6. Statistical analyses

All statistical analyses followed the official NHANES guidelines. WTMEC2YR was chosen as the weighting variable according to NHANES analysis guidelines, and final weights were determined based on the number of NHANES cycles (2011–2020), representing 21,911 U.S. individuals after weighting. Continuous variables were described using means and standard deviations (SD), while categorical variables were expressed as counts and weighted percentages. Between-group differences were assessed using chi-square tests or Student’s t tests. Multiple logistic regression analyses were conducted to examine the relationship between FLI and CKM syndrome, adjusting for potential confounders. Model 1 was a univariate analysis. Model 2 adjusted for age, sex, race/ethnicity, education and family income-to-poverty ratio. Model 3 additionally adjusted for drinking status, smoking status, hypertension, and diabetes. RCS analysis was employed to evaluate the nonlinear association between FLI and CKM syndrome risk. Subgroup analyses and interaction tests were used to explore the relationship between FLI and CKM syndrome risk in various populations. In addition, TyG, SII, and SIRI were used as mediating variables to evaluate the relationship between FLI and CKM prevalence.

Multiple imputation was applied for missing data to preserve sample size and avoid bias due to incomplete information.^[22]^ Data processing and analyses were performed using R software (version 4.3.0), with statistical significance set at a 2-sided *P*-value < .05^[23,24]^

## 3. Results

### 3.1. Participants’ characteristics based on CKM syndrome status

Table 1 summarizes the baseline characteristics of 8385 NHANES 2011–2020 participants (mean age 48.7 ± 17.6 years; 49.2% female). The 815 CKM syndrome patients (mean age 63.5 ± 14.3) exhibited distinct profiles: older age (vs 47.1 years), lower income (2.22 vs 2.54), higher hypertension prevalence (84.5% vs 15.5%), lower diabetes prevalence (42.7% vs 53.5%), and more frequent alcohol use (78.3% vs 21.7%). Elevated inflammatory markers (SII 604.9 vs 508.7; SIRI 1.56 vs 1.19; TyG 9.06 vs 8.67) and fatty liver index (76.7 vs 49.3) characterized CKM patients. In addition, CKM patients have lower levels of education and are mostly non-Hispanic.

**Table 1:** Characteristics of adult NHANES (2011–2020) participants with CKM data (N = 8385).

Characteristics	Overall (n = 8385)	No CKM (n = 7570)	CKM (n = 815)	*P*
Age (yr)*, mean ± SD	48.73 ± 17.55	47.13 ± 17.12	63.53 ± 14.28	<.001
Sex†, n (%)				<.001
Female	4123 (49.17)	3672 (48.51)	451 (55.34)	
Male	4262 (50.83)	3898 (51.49)	364 (44.66)	
Race/ethnicity†, n (%)				<.001
Mexican American	924 (11.02)	850 (11.23)	74 (9.08)	
Other Hispanic	766 (9.14)	698 (9.22)	68 (8.34)	
Non-Hispanic White	3599 (42.92)	3221 (42.55)	378 (46.38)	
Non-Hispanic Black	1890 (22.54)	1658 (21.90)	232 (28.47)	
Other race	1206 (14.38)	1143 (15.10)	63 (7.73)	
Education†, n (%)				<.001
<9th grade	615 (7.33)	511 (6.75)	104 (12.76)	
9–11th grade	1066 (12.71)	911 (12.03)	155 (19.02)	
High school diploma/GED	1832 (21.85)	1644 (21.72)	188 (23.07)	
Some College/AA degree	2647 (31.57)	2408 (31.81)	239 (29.33)	
≥College graduate	2225 (26.54)	2096 (27.69)	129 (15.83)	
Family income to poverty ratio^a^, mean ± SD	2.51 ± 1.66	2.54 ± 1.67	2.22 ± 1.53	<.001
BMI^a^, mean ± SD	29.05 ± 7.03	28.59 ± 6.84	33.37 ± 7.34	<.001
Drinking status†, n (%)				<.001
No	1287 (15.35)	1110 (14.66)	177 (21.72)	
Yes	7098 (84.65)	6460 (85.34)	638 (78.28)	
Smoking status†, n (%)				.111
No	6630 (79.07)	5968 (78.84)	662 (81.23)	
Yes	1755 (20.93)	1602 (21.16)	153 (18.77)	
Hypertension†, n (%)				<.001
No	5294 (63.14)	5168 (68.27)	126 (15.46)	
Yes	3091 (36.86)	2402 (31.73)	689 (84.54)	
Diabetes†, n (%)				<.001
No	7104 (84.72)	6668 (88.08)	436 (53.50)	
Yes	1060 (12.64)	712 (9.41)	348 (42.70)	
Borderline	221 (2.64)	190 (2.51)	31 (3.80)	
Triglycerides*	125.38 ± 82.75	121.95 ± 80.51	157.28 ± 95.53	<.001
High-density lipoprotein*	53.36 ± 11.48	53.99 ± 11.38	47.54 ± 10.69	<.001
Serum glucose*	107.68 ± 24.00	105.74 ± 20.73	125.66 ± 39.76	<.001
Serum creatinine*	0.92 ± 0.46	0.89 ± 0.36	1.25 ± 0.91	<.001
Gamma-glutamyl transpeptidase*	28.31 ± 38.94	27.02 ± 34.70	40.28 ± 65.27	<.001
eGFR*	93.78 ± 22.40	96.50 ± 19.97	68.48 ± 27.41	<.001
Albumin creatinine ratio*	44.35 ± 305.01	24.66 ± 221.66	227.20 ± 681.36	<.001
CKD†, n (%)				<.001
No	6900 (82.29)	6900 (91.15)	0 (0.00)	
Yes	1485 (17.71)	670 (8.85)	815 (100.00)	
CMS†, n (%)				<.001
No	5455 (65.06)	5455 (72.06)	0 (0.00)	
Yes	2930 (34.94)	2115 (27.94)	815 (100.00)	
TyG index*, Mean ± SD	8.71 ± 0.51	8.67 ± 0.48	9.06 ± 0.55	<.001
SII*, Mean ± SD	518.08 ± 317.82	508.73 ± 308.49	604.85 ± 383.61	<.001
SIRI*, Mean ± SD	1.23 ± 0.92	1.19 ± 0.89	1.56 ± 1.10	<.001
FLI*, Mean ± SD	51.99 ± 31.70	49.34 ± 31.39	76.66 ± 22.62	<.001

BMI = body mass index, CKD = chronic kidney disease, FLI = Fatty Liver Index, SD = standard deviation, SII = systemic immune-inflammation index, SIRI = systemic inflammation response index, TyG = triglyceride-glucose.

* Student *t* test.

† Chi-square test.

### 3.2. Relationship between FLI and CKM syndrome risk

Table 2 outlines the association between FLI and CKM syndrome risk. In the unadjusted model, FLI was positively associated with CKM syndrome risk (odds ratio [OR] = 1.03, 95% confidence interval [CI]: 1.03–1.04, *P* < .001). After adjusting for potential confounders in model 3, the association remained significant (adjusted OR = 1.03, 95% CI: 1.02–1.03, *P* < .001). This indicated a 3% increase in CKM syndrome risk for each unit increase in FLI. Participants were divided into quartiles based on FLI values. Compared to the first quartile, those in the second (adjusted OR = 4.81, 95% CI: 2.82–8.23, *P* < .001), third (adjusted OR = 8.99, 95% CI: 6.18–13.10, *P* < .001) exhibited progressively higher risks of CKM syndrome. The trend test across quartiles was significant (*P* < .001) (Table 2).

**Table 2:** Associations between FLI and CKM syndrome risk in adult participants of NHANES (2011–2020).

Variables	Model 1		Model 2		Model 3	
	OR (95% CI)	*P*	OR (95% CI)	*P*	OR (95% CI)	*P*
FLI	1.03 (1.03–1.04)	<.001	1.04 (1.03–1.04)	<.001	1.03 (1.02–1.03)	<.001
FLI group						
0.59–21.43	1.00 (Reference)		1.00 (Reference)		1.00 (Reference)	
21.43–51.54	7.77 (4.65–13.50)	<.001	4.61 (3.28–9.59)	<.001	4.81 (2.82–8.23)	<.001
51.54–81.70	16.10 (10.70–24.20)	<.001	12.40 (8.50–18.00)	<.001	8.99 (6.18–13.10)	<.001
81.70–100.00	34.20 (21.50–54.50)	<.001	32.70 (21.00–51.10)	<.001	18.10 (11.70–27.90)	<.001
*P* for trend	<.001		<.001		.001	

Model 1: Crude. Model 2: Adjust: Sex, Age, Race/ethnicity, Education, FIR. Model 3: Adjust: Sex, Age, Race/ethnicity, Education, FIR, Drinking status, Smoking status, Hypertension, Diabetes.

CI = confidence interval, OR = odds ratio.

### 3.3. Linear and nonlinear relationships between FLI and CKM syndrome risk

**Figure 2.**
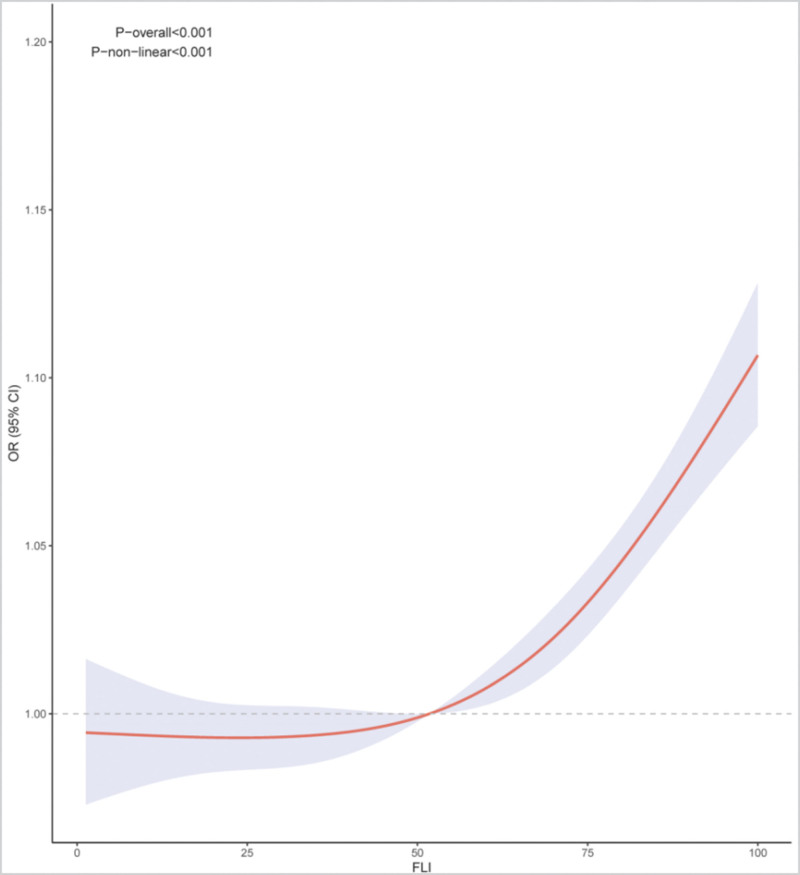
RCS analysis of the association between FLI and the risk of CKM syndrome in adult NHANES participants (2011–2020). CKM = cardiovascular-kidney-metabolic, FLI = Fatty Liver Index, NHANES = National Health and Nutrition Examination Survey, RCS = restricted cubic spline.

RCS analysis intuitively represents the relationship between FLI and CKM syndrome risk. Figure 2 shows that there is a nonlinear relationship between FLI and CKM syndrome risk after adjusting for confounding factors (p for nonlinear < 0.001, p for overall < 0.001). Threshold effect analysis further examined this nonlinear association, identifying an inflection point of 25.01 for FLI and CKM syndrome risk (Table 3). Below this threshold, FLI was positively and strongly associated with CKM syndrome risk, with a 12% increase in CKM syndrome risk for each unit increase in FLI (adjusted OR = 1.12, 95% CI: 1.05–1.21, *P* < .001). Above the threshold of 25.01, the relationship weakened but remained positive, with a 3% increase in CKM syndrome risk per unit increase in FLI (adjusted OR = 1.03, 95% CI: 1.02–1.03, *P* < .001).

**Table 3:** Threshold effect analysis of the relationship between FLI and CKM syndrome risk

	OR (95% CI)	*P*-value
One-line linear regression model	1.03 (1.02–1.03)	<.001
Two-piecewise linear regression model		
Inflection point	25.01	
<25.01	1.12 (1.05–1.21)	<.001
≥25.01	1.03 (1.02–1.03)	<.001
Log-likelihood ratio test		.008

CI = confidence interval, OR = odds ratio.

### 3.4. Subgroup analysis of FLI and CKM syndrome risk

**Figure 3.**
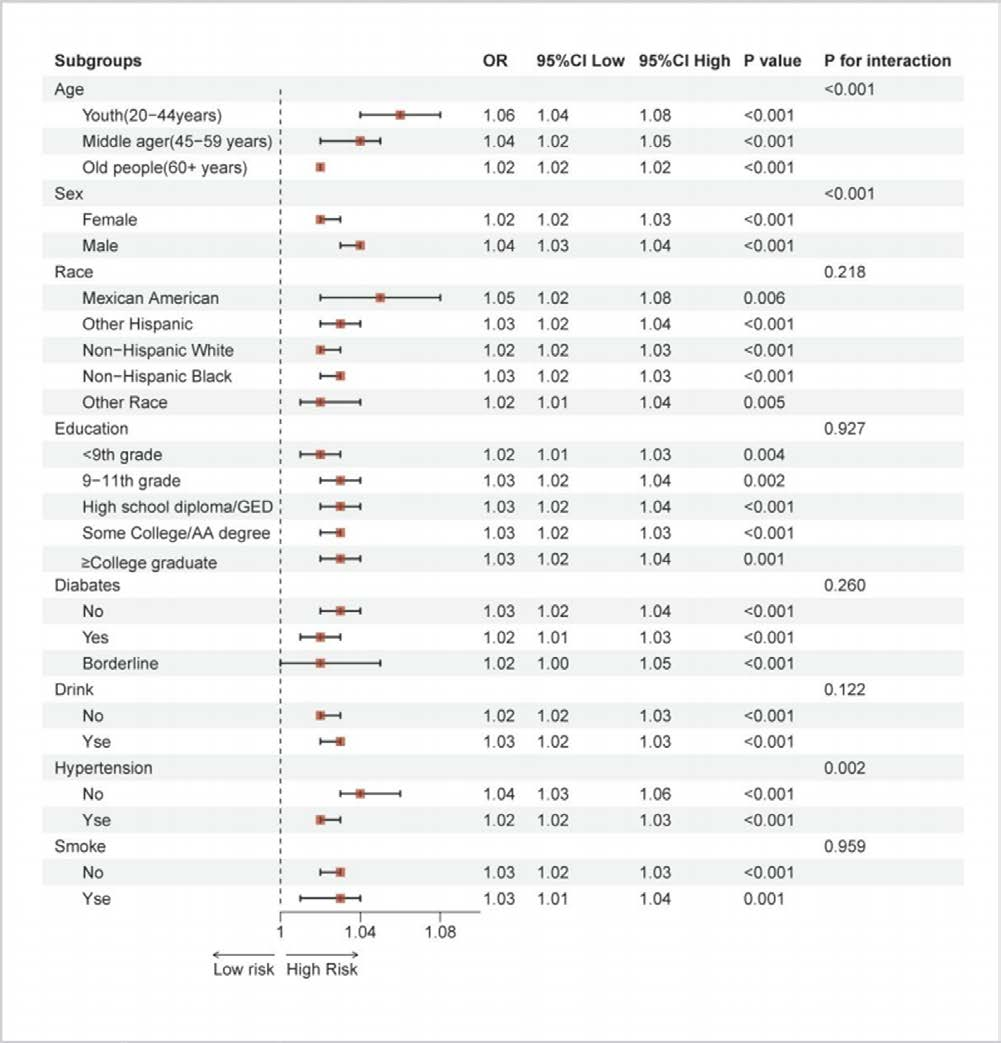
Subgroup analysis of FLI and risk of CKM syndrome. CKM = cardiovascular-kidney-metabolic, FLI = Fatty Liver Index.

Subgroup analyses assessed whether confounding factors influenced the relationship between FLI and CKM syndrome risk (Fig. 3). The positive association between FLI and CKM syndrome risk remained consistent across most populations, with no statistically significant associations observed in the Other Hispanic group or individuals with prediabetes. Interaction analysis revealed significant interactions between FLI and CKM syndrome risk concerning age (*P* for interaction = .002), gender (*P* for interaction < .001), and hypertension (*P* for interaction < .001).

**Figure 4.**
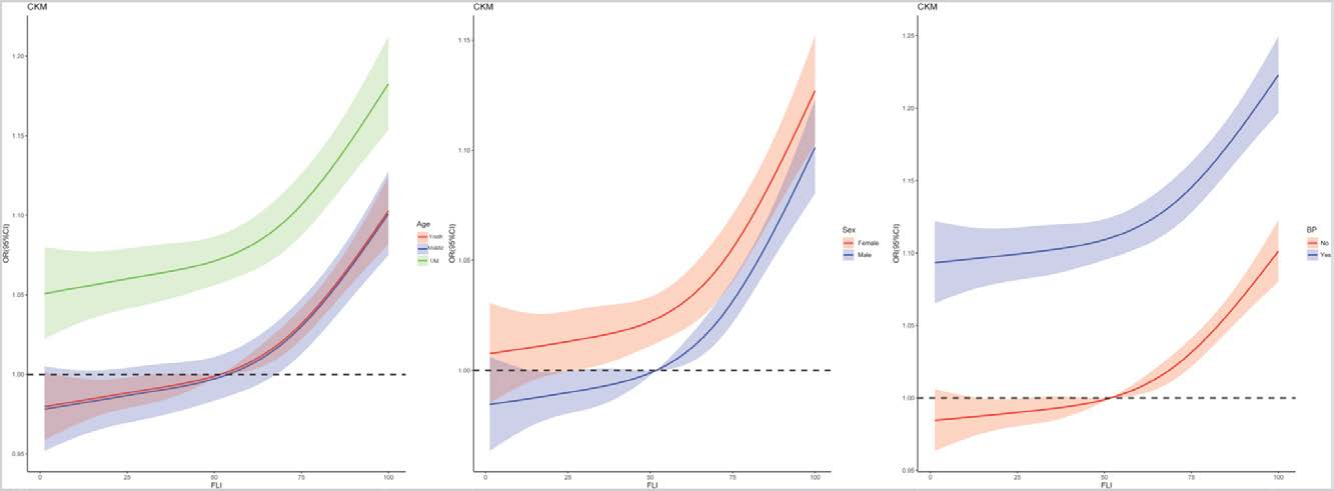
RCS analysis of the correlation between FLI and CKM syndrome risk in age, gender, and hypertension subgroups. CKM = cardiovascular-kidney-metabolic, FLI = Fatty Liver Index, RCS = restricted cubic spline.

The stratified RCS curve shows that the relationship between FLI and CKM is more significant in age, gender, and hypertension subgroups (Fig. 4).

### 3.5. The mediating effects of TyG, SII, and SIRI on the correlation between FLI and CKM incidence rates

**Figure 5.**
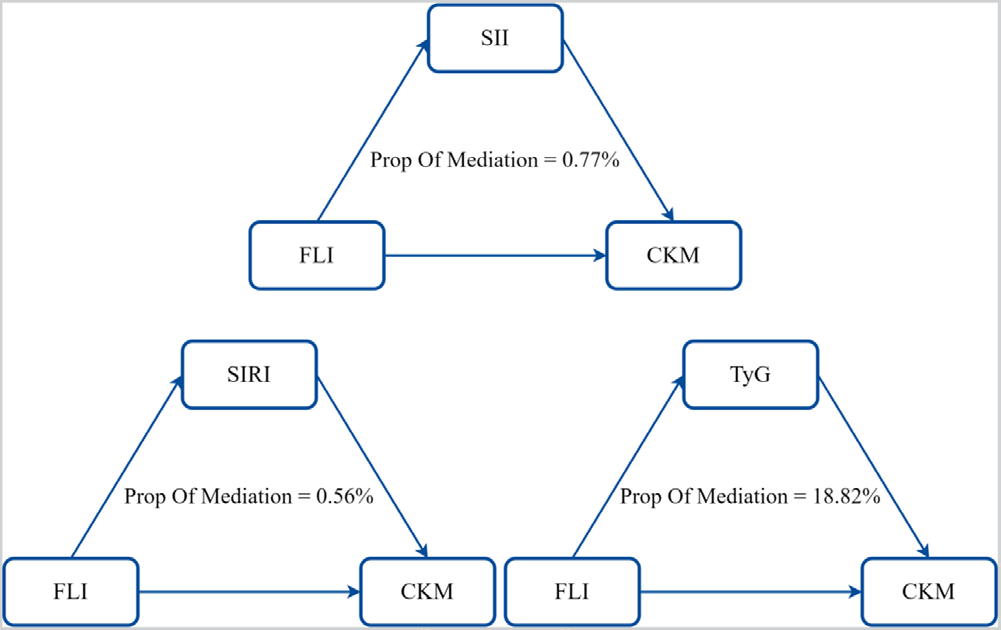
The mediating effects of TyG, SII, and SIRI on the correlation between FLI and CKM prevalence. CKM = cardiovascular-kidney-metabolic, FLI = Fatty Liver Index, SII = systemic immune-inflammation index, SIRI = systemic inflammation response index, TyG = triglyceride-glucose.

Mediation analysis showed that TyG, SII, and SIRI partially mediated the association between FLI and CKM incidence, with mediation ratios of 18.82%, 0.77%, and 0.56% (Fig. 5).

## 4. Discussion

This study analyzed 8385 U.S. adults and found a significant positive association between FLI and the risk of CKM syndrome. In the adjusted model, each unit increase in FLI was associated with a 3% increase in CKM syndrome risk. Participants in higher FLI quartiles had significantly elevated CKM syndrome risks compared to those in the lowest quartile, and this association remained significant after adjusting for potential confounders, such as demographic factors, lifestyle behaviors, and comorbidities. Subgroup analyses revealed that factors like age, gender, and hypertension modified this relationship. Additionally, a nonlinear association between FLI and CKM syndrome risk was observed, with a threshold effect around an FLI of 25.01. Beyond this point, the association weakened but remained statistically significant.

These findings align with previous research, which has consistently demonstrated a link between FLI and metabolic conditions, such as diabetes, cardiovascular disease, and CKD.^[25]^ However, this study extends the existing literature by exploring the relationship between FLI and the broader CKM syndrome spectrum. While earlier studies have suggested a dose-response relationship between FLI and metabolic syndrome, this study adds nuance by identifying a complex nonlinear relationship with a clear threshold effect.^[26–28]^

The association between FLI and CKM syndrome may be driven by factors such as chronic inflammation, oxidative stress, and dysregulation of the renin-angiotensin-aldosterone system. Evidence suggested that insulin resistance led to hepatic fat accumulation, resulting in NAFLD and increased risk of both cardiovascular and chronic kidney diseases.^[29,30]^ Chronic low-grade inflammation is a hallmark of fatty liver and other metabolic disorders. Obesity, for example, is characterized by a state of chronic inflammation, where adipose tissue releases pro-inflammatory cytokines (TNF-α, IL-6), which promote insulin resistance and atherosclerosis, both contributing to CKM syndrome.^[31,32]^ Furthermore, excessive oxidative stress can damage hepatocytes and disrupt vascular endothelial function, exacerbating the risk of metabolic and cardiovascular diseases.^[33,34]^

Furthermore, although the mediation effects of SII and SIRI were statistically significant, their contributions were relatively small (<1%), suggesting limited clinical relevance for these inflammatory indices in the pathway linking FLI and CKM syndrome. In contrast, the TyG index demonstrated a much larger mediating effect (18.82%), indicating that metabolic dysregulation rather than systemic inflammation may play a more dominant role in this association. These findings imply that the TyG index could serve as a more critical metabolic marker for CKM risk stratification and inform early prevention strategies.

Despite general consistency, our findings diverged from previous studies in several respects. Notably, after the FLI surpassed a threshold of 25.01, the relationship between FLI and CKM syndrome risk weakened and reached an equilibrium, although it remained positively correlated. In contrast, earlier research suggested a linear relationship between FLI and other metabolic diseases.^[35]^ This plateau effect at higher FLI levels may be attributed to several factors. One possibility is that an increase in FLI triggers inflammatory responses, which are later balanced by compensatory anti-inflammatory reactions as FLI surpasses a certain threshold.^[36–38]^ Additionally, inflammation-induced endothelial dysfunction may stabilize through vascular remodeling and adaptive responses.^[39]^ Increased insulin secretion and β-cell compensation might also explain the gradual stabilization of insulin resistance.^[40]^ Further research is necessary to elucidate the mechanisms underlying the nonlinear relationship between FLI and CKM syndrome risk.

Our stratified RCS analyses further clarified these interactions. Among older adults, the positive association between FLI and CKM risk was more pronounced, which may be attributed to cumulative metabolic burden and prolonged exposure to inflammatory stimuli, a phenomenon known as “inflammaging.”^[41]^ Regarding sex differences, males exhibited a stronger relationship, likely reflecting the influence of sex hormones such as lower estrogen levels, which are known to exert protective effects on lipid metabolism, vascular function, and inflammatory responses. Conversely, females may benefit from residual estrogen activity, particularly before menopause, which attenuates the adverse metabolic effects of hepatic steatosis. In participants with hypertension, the synergistic effects of hepatic fat accumulation and vascular dysfunction may amplify CKM risk. Hypertension promotes endothelial dysfunction, arterial stiffness, and activation of the renin–angiotensin–aldosterone system, which could interact with hepatic lipid accumulation to exacerbate cardiovascular and renal outcomes.^[42,43]^ Collectively, these findings suggest that the interplay between metabolic stress, hormonal environment, and vascular health modulates the strength of the FLI–CKM association across subgroups.

These findings suggest that FLI’s predictive value for CKM syndrome may vary across demographic and clinical subgroups, warranting further investigation into the modifying effects of these factors.

This study made 2 key contributions to the existing literature. First, it reinforced the utility of FLI as a predictor of CKM syndrome risk, underscoring its potential as a clinical screening tool.In practical terms, FLI can be easily implemented in clinical and primary care settings because it relies only on routine and widely available clinical parameters, namely BMI, waist circumference, triglycerides, and GGT. These measurements are inexpensive, noninvasive, and part of standard health examinations in most medical institutions. As such, FLI represents a cost-effective and feasible tool for early identification of individuals at high risk of CKM syndrome. Incorporating FLI into routine health checkups could help primary care physicians stratify risk, initiate lifestyle interventions earlier, and determine which patients may require more intensive metabolic or cardiovascular monitoring. While FLI had been widely used to assess fatty liver disease, diabetes, and other metabolic disorders, our findings suggested it might also be valuable in evaluating CKM syndrome risk, particularly in individuals with metabolic abnormalities. Second, this study advanced the understanding of metabolic syndrome by investigating the complex nonlinear relationship between FLI and CKM syndrome, with the identification of a threshold effect being particularly noteworthy. This provides new insights into how metabolic risk factors behave at different levels of liver fat.

However, several limitations must be acknowledged. First, the cross-sectional design of the NHANES data restricts the ability to infer causal relationships between FLI and CKM syndrome risk. Therefore, the present results should be interpreted as associations rather than evidence of causality. Although our findings suggest that higher FLI is linked to elevated CKM risk, they do not establish whether hepatic steatosis directly contributes to CKM development or merely reflects underlying metabolic dysfunction. From a clinical perspective, FLI may be considered a risk marker that helps stratify individuals at higher probability of CKM syndrome, but it cannot be used to infer disease causation. Future longitudinal studies and interventional trials are warranted to validate these associations, clarify temporal relationships, and determine whether FLI-guided prevention strategies could reduce cardiovascular and renal complications. Longitudinal studies are needed to determine whether FLI is a reliable predictor of CKM syndrome development over time. Second, although we adjusted for various confounders, residual confounding, particularly to dietary factors, genetic variations, and lifestyle behaviors, cannot be ruled out, as these variables were not fully captured in the dataset. Finally, the reliance on self-reported data for variables like alcohol consumption and smoking status introduces the potential for measurement bias. Future research should address these limitations by using more comprehensive and longitudinal datasets.

## 5. Conclusion

The study identified a significant positive association between the FLI and CKM syndrome risk. Importantly, this relationship exhibited a nonlinear positive correlation, with a plateau effect at higher FLI levels. These findings suggest that FLI could be a valuable tool for identifying individuals at elevated risk of CKM syndrome. However, to further validate the FLI-CKM syndrome relationship and clarify its causal nature, future longitudinal cohort studies are essential.

## Acknowledgments

We thank the National Center for Health Statistics for their efforts in creating the data for the NHANES.

## Author contributions

**Data curation**: Qinke Lv, Chun Yao.

**Funding acquisition**: Qinke Lv, Jian Zhong.

**Formal analysis**: Chun Yao.

**Methodology**: Chun Yao.

**Supervision**: Jian Zhong, Chun Yao.

**Validation**: Qinke Lv.

**Writing – original draft**: Qinke Lv.

**Writing – review & editing**: Qinke Lv.

## References

[1] Claudel SE Verma A. Albuminuria in cardiovascular, kidney, and metabolic disorders: a state-of-the-art review. Circulation. 2025;151:716–32. 40063723 10.1161/CIRCULATIONAHA.124.071079 PMC11902889

[2] Massy ZA Drueke TB. Combination of cardiovascular, kidney, and metabolic diseases in a syndrome named cardiovascular-kidney-metabolic, with new risk prediction equations. Kidney Int Rep. 2024;9:2608–18. 39291205 10.1016/j.ekir.2024.05.033 PMC11403032

[3] Zoccali C Vanholder R Massy ZA. The systemic nature of CKD. Nat Rev Nephrol. 2017;13:344–58. 28435157 10.1038/nrneph.2017.52

[4] Zhu R Wang R He J. Associations of cardiovascular-kidney-metabolic syndrome stages with premature mortality and the role of social determinants of health. J Nutr Health Aging. 2025;29:100504. 39952015 10.1016/j.jnha.2025.100504 PMC12180020

[5] Bedogni G Bellentani S Miglioli L. The fatty liver index: a simple and accurate predictor of hepatic steatosis in the general population. BMC Gastroenterol. 2006;6:33. 17081293 10.1186/1471-230X-6-33 PMC1636651

[6] Rinella ME Lazarus JV Ratziu V. A multisociety Delphi consensus statement on new fatty liver disease nomenclature. J Hepatol. 2023;79:1542–56. 37364790 10.1016/j.jhep.2023.06.003

[7] Pecani M Andreozzi P Cangemi R. Metabolic syndrome and liver disease: re-appraisal of screening, diagnosis, and treatment through the paradigm shift from NAFLD to MASLD. J Clin Med. 2025;14:2750. 40283580 10.3390/jcm14082750 PMC12028215

[8] Theodorakis N Nikolaou M. From cardiovascular-kidney-metabolic syndrome to cardiovascular-renal-hepatic-metabolic syndrome: proposing an expanded framework. Biomolecules. 2025;15:213. 40001516 10.3390/biom15020213 PMC11853431

[9] Lonardo A Nascimbeni F Ballestri S. Sex differences in nonalcoholic fatty liver disease: state of the art and identification of research gaps. Hepatology. 2019;70:1457–69. 30924946 10.1002/hep.30626 PMC6766425

[10] Bai Q Chen H Gao Z. Advanced prediction of heart failure risk in elderly diabetic and hypertensive patients using nine machine learning models and novel composite indices: insights from NHANES 2003-2016 [published online ahead of print February 27, 2025]. Eur J Prev Cardiol. doi: 10.1093/eurjpc/zwaf081. 10.1093/eurjpc/zwaf081 40036490

[11] Chen G Chen N Liu L. Correlation of serum cotinine with fatty liver index in adults: data from the NHANES March 2017 and 2018. BMC Gastroenterol. 2024;24:469. 39709381 10.1186/s12876-024-03572-6 PMC11663327

[12] Li B Liu Y Ma X Guo X. The association between non-high-density lipoprotein cholesterol to high-density lipoprotein cholesterol ratio and hepatic steatosis and liver fibrosis among US adults based on NHANES. Sci Rep. 2025;15:6527. 39988726 10.1038/s41598-025-90773-y PMC11847945

[13] Shi C Yuan C Hao Y Zhou Z Zhang Y. Association between surrogate indices of fatty liver and the risk of colorectal cancer: a cross-sectional United States study. Transl Cancer Res. 2025;14:313–26. 39974378 10.21037/tcr-24-1444 PMC11833385

[14] Gao C Gao S Zhao R. Association between systemic immune-inflammation index and cardiovascular-kidney-metabolic syndrome. Sci Rep. 2024;14:19151. 39160192 10.1038/s41598-024-69819-0 PMC11333479

[15] Ndumele CE Rangaswami J Chow SL. Cardiovascular-kidney-metabolic health: a presidential advisory from the American heart association. Circulation. 2023;148:1606–35. 37807924 10.1161/CIR.0000000000001184

[16] Grundy SM Brewer HB Cleeman JI Smith SC Lenfant C; American Heart Association. Definition of metabolic syndrome. Circulation. 2004;109:433–8. 14744958 10.1161/01.CIR.0000111245.75752.C6

[17] Levey AS Eckardt KU Tsukamoto Y. Definition and classification of chronic kidney disease: a position statement from kidney disease: improving global outcomes (KDIGO). Kidney Int. 2005;67:2089–100. 15882252 10.1111/j.1523-1755.2005.00365.x

[18] Levey AS Stevens LA Schmid CH. A new equation to estimate glomerular filtration rate. Ann Intern Med. 2009;150:604–12. 19414839 10.7326/0003-4819-150-9-200905050-00006 PMC2763564

[19] Gu X Gao D Zhou X. Association between fatty liver index and cardiometabolic multimorbidity: evidence from the cross-sectional national health and nutrition examination survey. Front Cardiovasc Med. 2024;11:1433807. 39301498 10.3389/fcvm.2024.1433807 PMC11411361

[20] Pei H Su X Wu S Wang Z. Evaluating the impact of chronic kidney disease and the triglyceride-glucose index on cardiovascular disease: mediation analysis in the NHANES. BMC Public Health. 2024;24:2750. 39385084 10.1186/s12889-024-20243-z PMC11462736

[21] He R Ye Y Zhu Q Xie C. Systemic immune-inflammation index is associated with high risk for prostate cancer among the U.S. elderly: evidence from NHANES 2001-2010. Front Oncol. 2024;14:1441271. 39376981 10.3389/fonc.2024.1441271 PMC11456397

[22] Jiang J Zhao H Chen J. The association between dietary creatine intake and cancer in U.S. adults: insights from NHANES 2007-2018. Front Nutr. 2024;11:1460057. 39867555 10.3389/fnut.2024.1460057 PMC11757134

[23] Rammon J He Y Parker JD. Multiple imputation to account for linkage ineligibility in the NHANES-CMS Medicaid linked data: general use versus subject specific imputation models. Stat J IAOS. 2019;35:443–56. 32831968 10.3233/sji-180470 PMC7437981

[24] Mao Q Zhu X Kong Y. Sleep duration mediates the association between heavy metals and the prevalence of depression: an integrated approach from the NHANES (2005-2020). Front Psychiatry. 2024;15:1455896. 39286395 10.3389/fpsyt.2024.1455896 PMC11404323

[25] Mao Q Zhu X Zhang X Kong Y. Triglyceride-glucose index and its combination with obesity indicators mediating the association between 2-hydroxyfluorene and the prevalence of cardiovascular disease: evidence from the NHANES (2005-2018). Ecotoxicol Environ Saf. 2024;287:117283. 39504874 10.1016/j.ecoenv.2024.117283

[26] Zhang R Ren S Mi H. Fatty liver index as an independent predictor of all-cause and disease-specific mortality. Eur J Gastroenterol Hepatol. 2024;36:1453–63. 39400538 10.1097/MEG.0000000000002865 PMC11527378

[27] Álvares-da-Silva MR Vargas MDS Rabie SMS. FLI and FIB-4 in diagnosing metabolic dysfunction-associated steatotic liver disease in primary care: high prevalence and risk of significant disease. Ann Hepatol. 2025;30:101584. 39395769 10.1016/j.aohep.2024.101584

[28] Li N Li Y Cui L. Association between different stages of cardiovascular-kidney-metabolic syndrome and the risk of all-cause mortality. Atherosclerosis. 2024;397:118585. 39255681 10.1016/j.atherosclerosis.2024.118585

[29] Chen Y Zhao X. The mediating role of insulin resistance in the association between inflammatory score and MAFLD: NHANES 2017-2018. Immun Inflamm Dis. 2024;12:e70035. 39364712 10.1002/iid3.70035 PMC11450453

[30] Kim B Ronaldo R Kweon BN. Mesenchymal stem cell-derived exosomes attenuate hepatic steatosis and insulin resistance in diet-induced obese mice by activating the FGF21-adiponectin axis. Int J Mol Sci. 2024;25:10447. 39408777 10.3390/ijms251910447 PMC11476820

[31] Baj J Kołodziej M Kobak J. Significance of immune and non-immune cell stroma as a microenvironment of hepatocellular carcinoma-from inflammation to hepatocellular carcinoma progression. Int J Mol Sci. 2024;25:10233. 39408564 10.3390/ijms251910233 PMC11475949

[32] Mori T Yoshio S Kakazu E Kanto T. Active role of the immune system in metabolic dysfunction-associated steatotic liver disease. Gastroenterology Report. 2024;12:goae089. 39411101 10.1093/gastro/goae089 PMC11479709

[33] Minetti ET Hamburg NM Matsui R. Drivers of cardiovascular disease in metabolic dysfunction-associated steatotic liver disease: the threats of oxidative stress. Front Cardiovasc Med. 2024;11:1469492. 39411175 10.3389/fcvm.2024.1469492 PMC11473390

[34] Wan W Wei R Xu B. Qiwei Jinggan Ling regulates oxidative stress and lipid metabolism in alcoholic liver disease by activating AMPK. Phytomedicine 2024;135:156125. 39388920 10.1016/j.phymed.2024.156125

[35] Zhang F Han Y Wu Y. Association between triglyceride glucose-body mass index and the staging of non-alcoholic steatohepatitis and fibrosis in patients with non-alcoholic fatty liver disease. Ann Med. 2024;56:2409342. 39348274 10.1080/07853890.2024.2409342 PMC11443541

[36] Hyun YY. Obesity and chronic kidney disease, an important piece in the puzzle of Cardiovascular-kidney- metabolic syndrome. Korean J Intern Med. 2024;39:700–1. 39252491 10.3904/kjim.2024.273 PMC11384253

[37] Duan S Tu Z Duan L Tu R. Differential effects of systemic immune inflammation indices on hepatic steatosis and hepatic fibrosis: evidence from NHANES 1999-2018. BMC Gastroenterol. 2024;24:463. 39695411 10.1186/s12876-024-03557-5 PMC11658444

[38] Wang Y Chen S Tian C. Association of systemic immune biomarkers with metabolic dysfunction-associated steatotic liver disease: a cross-sectional study of NHANES 2007-2018. Front nutr. 2024;11:1415484. 39296508 10.3389/fnut.2024.1415484 PMC11408230

[39] Ghorbanpour S Cartland SP Chen H. The FKBPL-based therapeutic peptide, AD-01, protects the endothelium from hypoxia-induced damage by stabilising hypoxia inducible factor-α and inflammation. J Transl Med. 2025;23:309. 40069829 10.1186/s12967-025-06118-w PMC11895374

[40] Kanbay M Copur S Guldan M Ozbek L Mallamaci F Zoccali C. Glucagon and glucagon-like peptide-1 dual agonist therapy: a possible future towards fatty kidney disease. Eur J Clin Invest. 2025;55:e14330. 39400355 10.1111/eci.14330

[41] Franceschi C Garagnani P Parini P Giuliani C Santoro A. Inflammaging: a new immune-metabolic viewpoint for age-related diseases. Nat Rev Endocrinol. 2018;14:576–90. 30046148 10.1038/s41574-018-0059-4

[42] Regitz-Zagrosek V. Sex and gender differences in health. Science & Society Series on Sex and Science. EMBO Rep. 2012;13:596–603. 22699937 10.1038/embor.2012.87 PMC3388783

[43] Guldan M Unlu S Abdel-Rahman SM. Understanding the role of sex hormones in cardiovascular kidney metabolic syndrome: toward personalized therapeutic approaches. J Clin Med. 2024;13:4354. 39124622 10.3390/jcm13154354 PMC11312746

